# Phyllodes tumor of the verumontanum: a case report

**DOI:** 10.1186/s13000-015-0314-0

**Published:** 2015-06-16

**Authors:** Jin Tang, Leye He, Zhi Long, Jingchao Wei

**Affiliations:** Department of Urology, The Third Xiangya Hospital of Central South University, Changsha, 410013 China

**Keywords:** Phyllodes tumor, Verumontanum, Treatment

## Abstract

**Abstract:**

The current report presents the case of a 42-year-old male with extraordinarily salient urination difficulty that had lasted 6 months. Transrectal ultrasonography and pelvic magnetic resonance imaging demonstrated prostatic hyperplasia and cyst. PSA level was 20.65 (>4) μg/L in the patient. Transrectal prostatic biopsy revealed benign prostatic hyperplasia. He agreed to receive plasmakinetic resection of the prostate. During operation a lobulated lump was unexpectedly found on the verumontanum, with the prostate macroscopically normal. Complete tumor excision was performed and pathological assessment indicated phyllodes tumor of the verumontanum. The patient had an uneventful post-operative course and recovered well. The diagnosis, histological classification, treatment, and prognosis of this case are presented. It is necessary to perform cystoscopy to exclude verumontanum tumor even when all imaging examinations indicate prostate hyperplasia, especially in young males.

**Virtual Slides:**

The virtual slide(s) for this article can be found here: http://www.diagnosticpathology.diagnomx.eu/vs/1868931661161758

## Background

The incidence of verumontanum tumor is low. Several phyllodes tumors of the seminal vesicle and prostate have been reported; however, studies describing phyllodes tumor of the verumontanum are scarce.

**Fig. 1:**
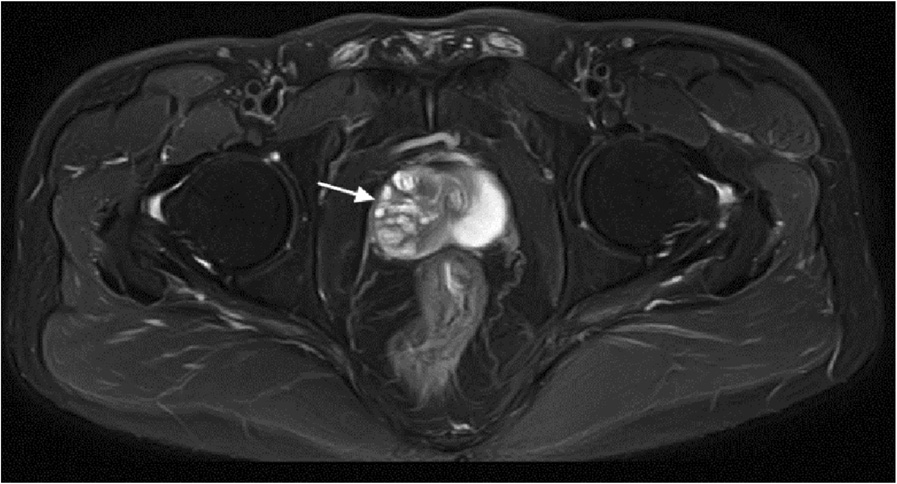
MRI—Transverse section. T2 weighted phase. Multiple nodules of long T2 signal are visible in the right area of the prostate

**Fig. 2:**
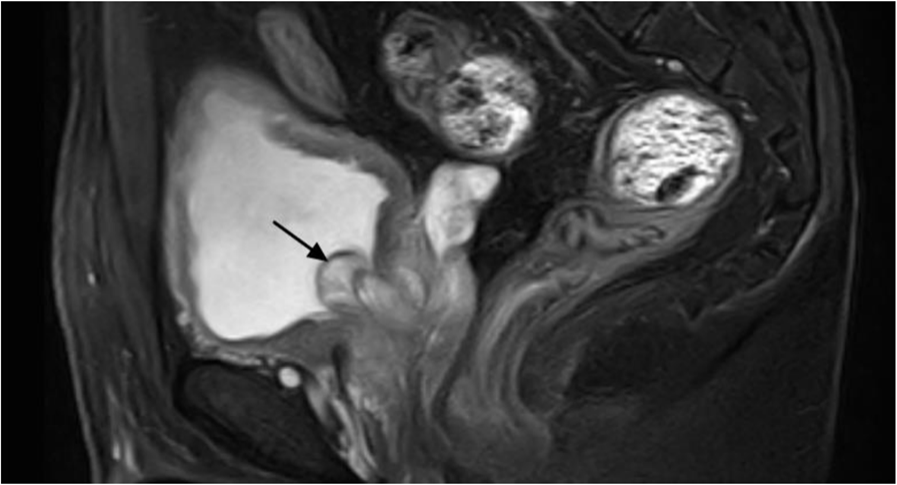
MRI—Sagittal section. T2 weighted phase. Bump of posterior urethral extruding to the bladder

**Fig. 3:**
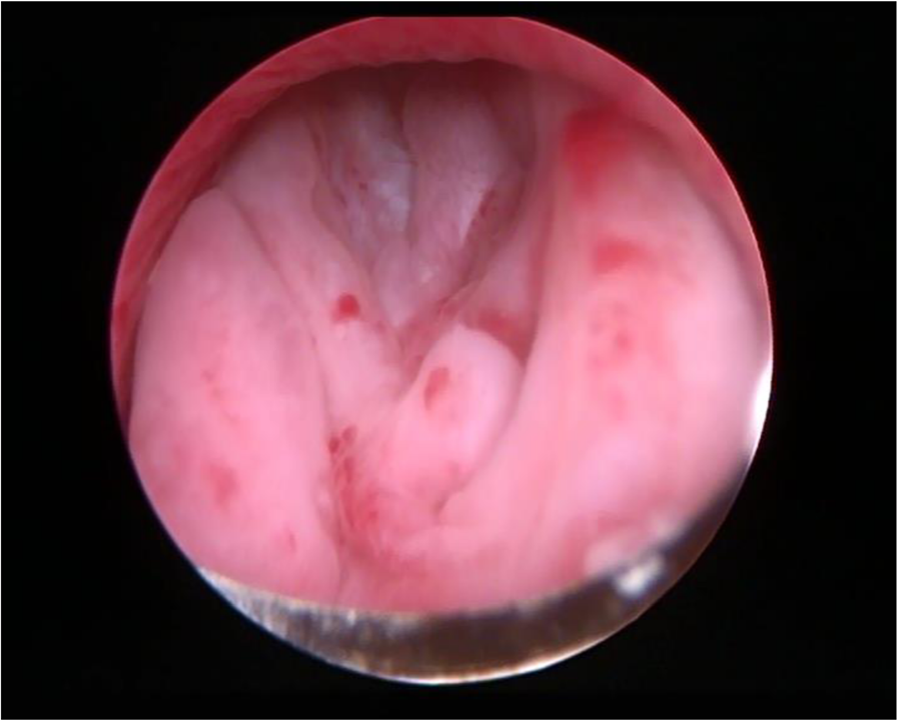
Intraoperative view. A papillary lobulated lump with intact surface was found during the operation, acting as a valve for the bladder outlet

**Fig. 4:**
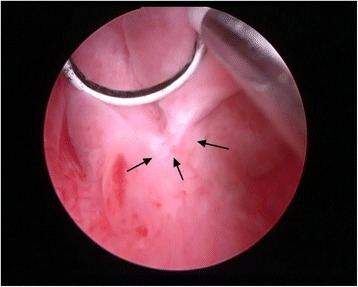
Intraoperative view. The lump was connected to the verumontanum by a pedicle

**Fig. 5:**
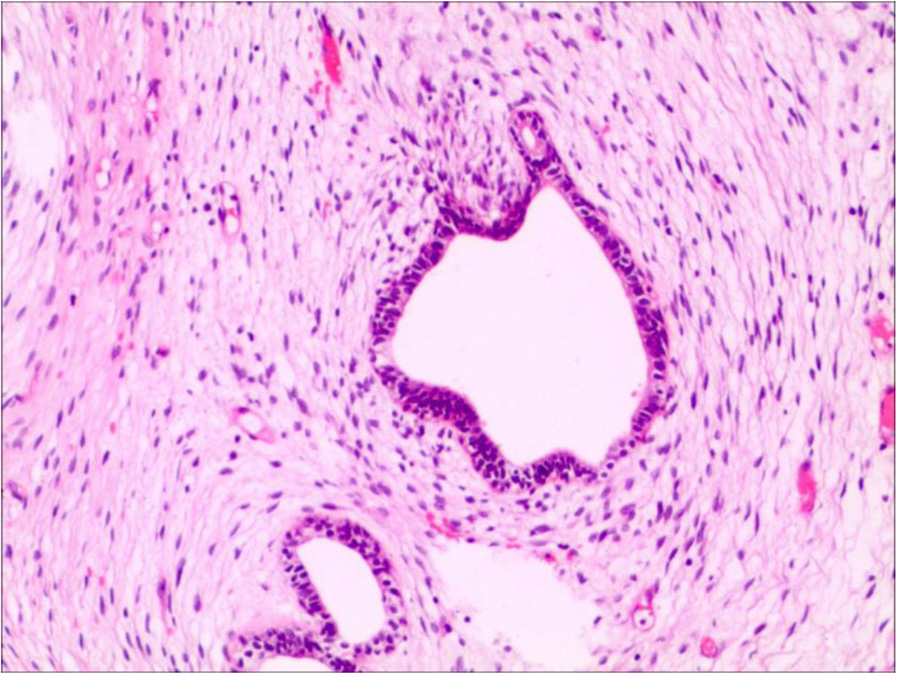
Microscopic assessment of the tumor revealed an epithelial-stromal tumor, including irregular-shaped benign glandular elements and excessive hyperplasia of stromal tumor cells. Stromal cells exhibit pleomorphism, and were multinucleated and giant (hematoxylin and eosin; original magnification × 200)

**Fig. 6:**
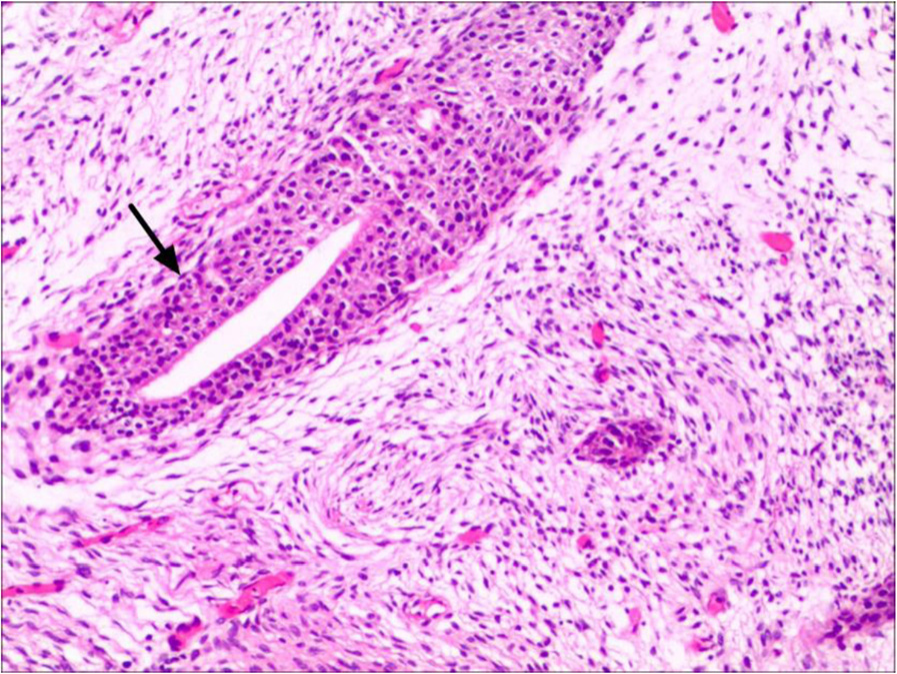
An irregular cleft like space is lined by epithelium and invested with cellular stroma (H & E, original magnification × 200)

**Fig. 7:**
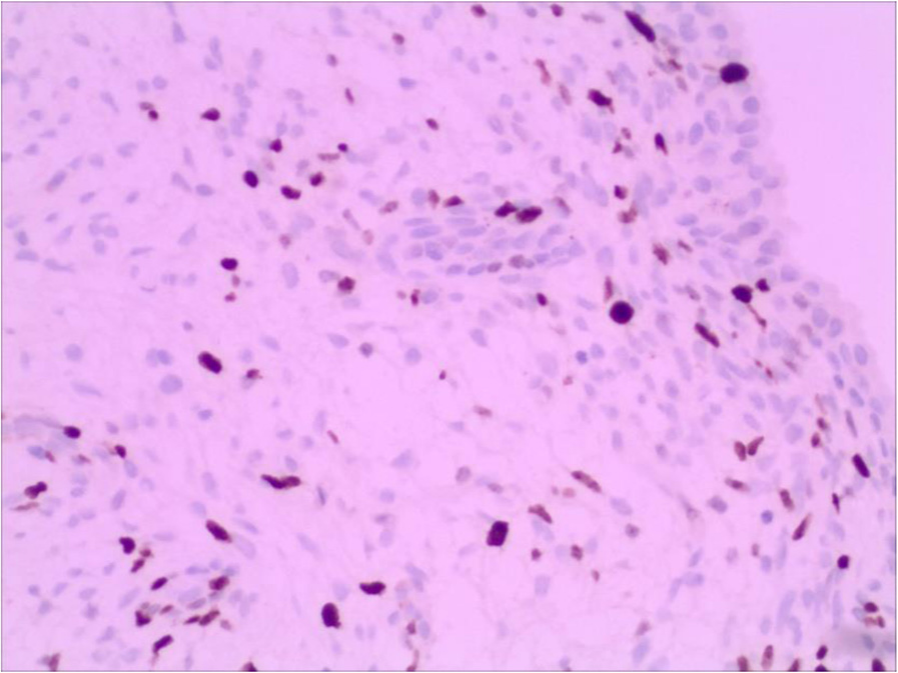
A total of 500 cells were counted at the most intense Ki-67-stained area for the assessment of Ki-67 positivity(100 × magnification)

Phyllodes tumor is defined as a neoplasm with a foliated structure composed of cellular stroma and benign epithelial elements. Most phyllodes tumors occur in the breast [1]. Here, a patient with a phyllodes tumor of the verumontanum is reported, and the histological classification, treatment, and prognosis of the tumor are discussed.

## Case presentation

In December 2014, a 42-year-old male presented to the Department of Urology, The Third Xiangya Hospital of Central South University (Changsha, China) with a six-month history of dysuria and urgent urination. He did not report frequent urination or gross hematuria. Digital Rectal Examination revealed a hard nodule in the right posterior part of the prostate. No other anomaly was observed after physical examination. The maximum urine flow rate was 6 ml/s, and urodynamic examination indicated bladder outlet obstruction. The patient had no significant medical history. Serum prostate-specific antigen (PSA) levels were 21 ng/mL. Transrectal ultrasonography disclosed a serious possibility of prostatic hyperplasia and cyst. Pelvic magnetic resonance imaging revealed a hyperplastic nodule with cystic changes and a clear boundary Figs. 1 and 2. An 8-core transrectal ultrasound-guided prostate biopsy was performed. Histopathology revealed benign prostatic hyperplasia. The patient agreed to undergo plasmakinetic resection of the prostate. During the surgey a lobulated lump with intact external surface was unexpectedly found on the verumontanum, to which it was connected by pedicle. The lump constituted a valve for posterior urethra, resulting in bladder outlet obstruction and difficult urination Figs. 3 and 4. There was no obvious hyperplasia of the prostate. Complete lump excision was then performed and pathological assessments revealed phyllodes tumor of the verumontanum. The lump surface was coated with transitional epithelium, and a significant hyperplasia of fibers and fibroblasts in its mesenchyme was observed. Scattered myxoid degeneration was also found Figs. 5 and 6. The expression of Ki-67 was evaluated by immunohistochemical staining, and a Ki-67 proliferation index of about 12 % was obtained (Fig. 7). The patient had an uneventful post-operative course and recovered well. The maximum flow rate examined after the operation was 23 mL/s.

## Discussion

To our knowledge, no previous reports of verumontanum phyllodes tumor have been published. This may be the first case of phyllodes tumor of the verumontanum. Previously reported phyllodes tumors are mostly located in the female breast tissue, followed by the prostate [2]. Less than 15 and 100 cases of phyllodes tumors of the seminal vesicle and prostate, respectively, have been reported to date. Verumontanum tumors are rare, with extremely atypical symptoms. Phyllodes tumors are rare even in the breast, where they account for less than 1 % of all neoplasms [1]. Ferrariet et al. proposed that phyllodes tumor of the prostate is a potential diagnosis for previously healthy young men with hydronephrosis and lower urinary tract symptoms [3]. The patient described here presented with salient difficult urination. Intraoperative observation revealed overt hyperplasia of the bladder’s trabecula and diverticulum. Image assessment also indicated prostatic hyperplasia.

The concept of phyllodes tumor was initially used for breast tissues. In 1982, the World Health Organization (WHO) declared the term “phyllodes tumor” to be the most appropriate among more than 60 synonyms of breast neoplasms [4]. Compared to breast adenocarcinoma, phyllodes tumors tend to affect younger individuals [5]. The majority of phyllodes tumors of the breast have infiltrative margins. Phyllodes tumors are epithelial stromal tumors, with the unique feature of being a blend of stromal and epithelial tissues; the presence of both epithelial and stromal elements is necessary to confirm the diagnosis [2]. The misdiagnosis rate of breast phyllodes tumors is relatively high, since they are histologically similar to fibroadenoma. It is important to differentiate phyllodes tumors from other benign breast tumors [1]. The accepted histological classification of phyllodes tumors in the breast is to divide masses into benign, borderline, and malignant subtypes according to features such as tumor margins (pushing or infiltrative), stromal overgrowth, tumor necrosis, cellular atypia, and the number of mitotic cells per high power field [2, 4, 6]. No cases of phyllodes tumor of the verumontanum have been reported, and it is difficult to develop any definitive criteria for its grading. In the patient described here, an estimated Ki-67 proliferation index of 12 % was obtained. This finding indicated that tumor cell proliferation was not active, developing relatively slowly. In the mammary gland, the presence of high cellularity and mitotic activity correlates with tumors of malignant and metastasizing potential [7].

Few reliable clinical and histological prognostic factors of phyllodes tumors have been identified. Fain JS et al. reported the first case of high-grade phyllodes tumor of the seminal vesicle, with a lung metastasis four years after cystoprostatoseminovesiculectomy. They proposed that like breast and prostate tumors, phyllodes tumors of the seminal vesicle should be considered high-grade lesions (malignant) with significant mitotic activity, stromal pleomorphism, and stromal overgrowth [8]. Abe et al. reported a patient suffering from cystosarcoma phyllodes of the seminal vesicle who died 11 months after tumor removal by open operation [9]. The selection of the initial surgical approach is of great importance for phyllodes tumors of the breast. Either wide excision or mastectomy is recommended for breast phyllodes tumors, provided histologically clear specimen margins are ensured [1]. Complete surgical resection offers high rates of local control and disease-free survival [4]. However, in rare cases where metastasis occurs, prognosis tends to be poor [5]. Complete excision of the tumor is appropriate for its intact surface, and the patient described here had a quick recovery.

## Conclusion

A rare case of phyllodes tumor of the verumontanum was described in this report. This neoplasm had similar symptoms with prostatic hyperplasia, and was misdiagnosed before surgery. For young males, posterior urethral tumors are potential diagnoses when they present with salient symptoms of bladder outlet obstruction. Phyllodes tumor of the verumontanum, as seen with this case, has possible local proliferative activity without metastatic potential, and transurethral resection may be required. Close periodical follow-up is essential, as progression requiring more aggressive surgical intervention can be observed.

## Consent

Written informed consent was obtained from the patient for publication of this Case Report and any accompanying images. A copy of the written consent is available for review by the Editor-in-Chief of this journal.
